# Generating real-world evidence from unstructured clinical notes to examine clinical utility of genetic tests: use case in BRCAness

**DOI:** 10.1186/s12911-020-01364-y

**Published:** 2021-01-06

**Authors:** Yiqing Zhao, Saravut J. Weroha, Ellen L. Goode, Hongfang Liu, Chen Wang

**Affiliations:** 1 Division of Digital Health Sciences, Department of Health Sciences Research, Mayo Clinic, 205 3rd Ave SW, Rochester, MN 55905 USA; 2 Division of Medical Oncology, Department of Oncology, Mayo Clinic, 200 1st St SW, Rochester, MN 55905 USA; 3 Division of Epidemiology, Department of Health Sciences Research, Mayo Clinic, 205 3rd Ave SW, Rochester, MN 55905 USA; 4 Division of Biomedical Statistics and Informatics, Department of Health Sciences Research, Mayo Clinic, 205 3rd Ave SW, Rochester, MN 55905 USA

**Keywords:** Precision medicine, Real-world evidence, BRCA1/2, PARP inhibitor, Electronic health records, Natural language processing

## Abstract

**Background:**

Next-generation sequencing provides comprehensive information about individuals’ genetic makeup and is commonplace in oncology clinical practice. However, the utility of genetic information in the clinical decision-making process has not been examined extensively from a real-world, data-driven perspective. Through mining real-world data (RWD) from clinical notes, we could extract patients’ genetic information and further associate treatment decisions with genetic information.

**Methods:**

We proposed a real-world evidence (RWE) study framework that incorporates context-based natural language processing (NLP) methods and data quality examination before final association analysis. The framework was demonstrated in a Foundation-tested women cancer cohort (N = 196). Upon retrieval of patients’ genetic information using NLP system, we assessed the completeness of genetic data captured in unstructured clinical notes according to a genetic data-model. We examined the distribution of different topics regarding *BRCA1/2* throughout patients’ treatment process, and then analyzed the association between *BRCA1/2* mutation status and the discussion/prescription of targeted therapy.

**Results:**

We identified seven topics in the clinical context of genetic mentions including: Information, Evaluation, Insurance, Order, Negative, Positive, and Variants of unknown significance. Our rule-based system achieved a precision of 0.87, recall of 0.93 and F-measure of 0.91. Our machine learning system achieved a precision of 0.901, recall of 0.899 and F-measure of 0.9 for four-topic classification and a precision of 0.833, recall of 0.823 and F-measure of 0.82 for seven-topic classification. We found in result-containing sentences, the capture of *BRCA1/2* mutation information was 75%, but detailed variant information (e.g. variant types) is largely missing. Using cleaned RWD, significant associations were found between *BRCA1/2* positive mutation and targeted therapies.

**Conclusions:**

In conclusion, we demonstrated a framework to generate RWE using RWD from different clinical sources. Rule-based NLP system achieved the best performance for resolving contextual variability when extracting RWD from unstructured clinical notes. Data quality issues such as incompleteness and discrepancies exist thus manual data cleaning is needed before further analysis can be performed. Finally, we were able to use cleaned RWD to evaluate the real-world utility of genetic information to initiate a prescription of targeted therapy.

## Background

*BRCA1/2* germline mutations are considered a risk factor for breast, ovarian, and other hereditary cancers [1, 2]. *BRCA1/2* plays an important role in the maintenance of genome integrity and DNA repair through homologous recombination repair (HRR) pathway [3]. Poly-adenosyldiphosphate-ribose polymerase (PARP) inhibitors are a relatively new type of cancer treatment initially designed to target HRR defects, especially for people with inherited mutations in *BRCA1/2 *[4]. Recent studies prove that mechanisms and treatment susceptibility of BRCA-mutant tumors are not restricted to inherited tumor – both familial and sporadic tumor share common clinical features [5]: extreme levels of genomic instability, basal-like transcriptomic signature (genes expression profile similar to normal breast myoepithelial layer), and triple-negative phenotype (oestrogen receptor, progesterone receptor and *ERBB2* oncogene not expressed or amplified). With refined knowledge of the biological mechanism of *BRCA1/2* tumor suppressor functions over the past decade, the concept of “BRCAness” was introduced as: “A phenocopy of BRCA1 or BRCA2 mutation; it describes the situation in which an HRR defect exists in a tumor in the absence of a germline BRCA1 or BRCA2 mutation.” The new understanding of BRCAness has driven wider adoption of Precision Medicine approaches, PAPR inhibitor specifically, to treat sporadic BRCA-mutant cancer.

The potential of Precision Medicine is to enable medical practitioners to make better clinical decisions by incorporating individual patients’ genomic information and clinical characteristics, to improve the selection of targeted therapies, avoid side effects from ineffective therapy, and therefore achieve desirable cost-effectiveness [6–9]. Due to the heterogeneity of different malignancies and diverse treatment considerations, patient size for similar clinical characteristics (e.g. receiving the same therapies, with similar disease stages, and have similar tumor tissue genetic makeup) is often small and makes it especially difficult for conducting clinical trial research for Precision Medicine therapies. To facilitate effective implementation of Precision Medicine and evaluations of its clinical benefits, it has become increasingly important to leverage real-world data (RWD) and generate real-world evidence (RWE) to understand the challenges of maximizing clinical benefits of individual cancer patients, given knowledge of individual tumor’s characterization and options of new targeted therapies. Ideally, successful utilization of RWD will not only assist evaluations of Precision Medicine clinical utilities but also help to make novel discoveries for further advancing Precision Medicine applications.

Genetic testing (germline or somatic) involves examining a person’s DNA [10] and can reveal mutations in genes that may contribute to an increased risk of disease (predictive genetic tests [11]) or a different response to therapies (pharmacogenomics [12]). The advancement of next-generation sequencing and genetic testing has played an increasingly important role in the practice of Precision Medicine [13–15]. However, the clinical utility of genetic testing remains unevaluated in the real-world setting. With the wide adoption of electronic health records (EHRs), we can make secondary use of data from EHRs to study clinical questions in a real-world setting [16–19]. To examine how genetic data was utilized in the clinical decision-making processes, we first need to extract and curate genetic information as well as other associated clinical information from EHRs. Successful retrieval of comprehensive information will enable evaluation of (1) utilization and the utility of genetic tests results in clinical decision making, (2) different cancer subtypes’ susceptibility to targeted therapies, (3) potential long-term benefit of genetic tests, and relevant targeted therapies in overall survival.

Despite the premise of using RWD for advancing Precision Medicine, data quality significantly limits the usages of RWD for RWE studies [20–22]. For example, the missingness of RWD from current structured electronic health records (EHRs) is one major concern [23]. As part of our prior research [24], we have examined EHRs at Mayo Clinic and found it challenging to completely capture genetic information generated by diverse commercial vendors in a structured EHR system. Natural language processing (NLP) techniques have been used to extract data from unstructured clinical notes and have been applied to the extraction of various clinical data elements such as disease phenotypes [25], adverse drug events [26], lab test results [27], and recently HLA genotypes [28]. Current work that utilized NLP for Precision Medicine studies emphasized clinical phenotype extraction while structured genetic information was already available from sequencing labs directly [29, 30] or from a structured genetic database linked to the EHR system [31]. However, given the substantial heterogeneity in how genetic information was documented in EHR systems [32], extracting/utilizing genetic information from unstructured clinical notes for research reuses has been of pressing need. Related to this proposed work, there were only a few previous efforts to extract genetics data from unstructured EHRs: for example, Lee et al. have attempted to extract HLA genotype information from free-text clinical notes using rule-based methods, yet, restricting to a limited number of HLA-related variants [28]. Guan et al. classified progress reports of 755 cancer patients to the treatment-change and no-treatment-change groups, using NLP keyword matching and recurrent neural network (RNN) [33]; however, no genetic information was extracted in this works thus undermined the interpretability of their model. Although some previous works have been done to reuse genetic information in clinical notes, the generalization and interpretability of these works are limited. To address these challenges, we proposed an RWE study framework that incorporates context-based NLP methods for data (genetic information) extraction and data quality examination before final classification and association analysis was performed. The novelty of our work is that we were able to extract patients’ personal genetic information by distinguishing it from general genetic information also documented in EHRs. Therefore, we can guarantee the data quality of genetic information we extracted from unstructured EHRs and enhance the interpretability of our analysis model when using it as features for downstream analysis.

## Methods

To conduct RWE research of the utility of BRCAness in a clinical setting, we started with constructing a cohort and corpus with comprehensive genetic and clinical information available from different linked sources (“Cohort and corpus” section). With the awareness of contextual variability in clinical notes, NLP-based approaches were applied to identify “topics” in clinical notes regarding *BRCA1/2* mentions (“Sentence extraction and topic identification” section). After defining relevant topics, we developed and compared the rule-based and machine learning NLP system for genetic information extraction (“NLP system development and evaluation” section). We visualized and investigated temporal distributions of topic occurrences across patients’ medical journeys (“Rule-based system”). Data quality issues such as incompleteness and discrepancy were examined (“Temporal examination of topics and data quality examination” section). Finally, we were able to use cleaned RWD to conduct a real-word evidence study regarding the association between *BRCA1/2* mutation and prescription of PARP inhibitors (“Association analysis between mutation and targeted therapy” section).

### Cohort and corpus

Our cohort included 196 women cancer (breast, ovary, cervix, and uterus) patients that have conducted Foundation Medicine genetic tests with reports returned back to Mayo Clinic. Foundation Medicine, Inc. offered somatic genetic tests to qualifying patients across all solid tumors. With research authorization, we collected their genetic reports from Foundation Medicine, Inc. as well as unstructured clinical notes until March 31, 2020 from Mayo Clinic clinical data warehouse. The data warehouse integrated clinical notes from Mayo Clinic historical notes (Minnesota, Arizona, Florida, Mayo Clinic Health System notes), and Mayo Clinic Epic notes (Minnesota from May 2018, Arizona, Florida from Oct 2018, Mayo Clinic Health System from 2017). This research project was reviewed by the Mayo Clinic Institutional Review Board.

### Sentence extraction and topic identification

**Fig. 1:**
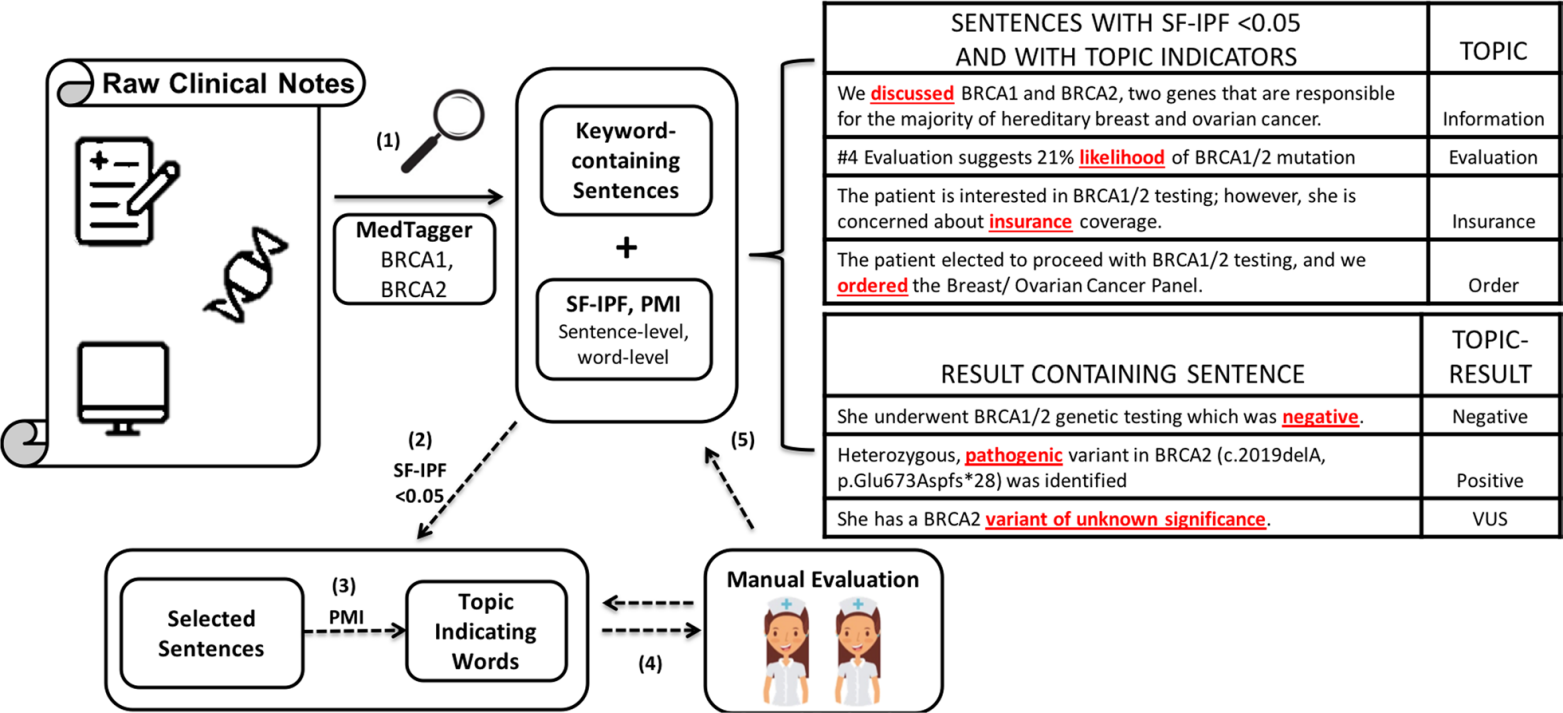
Workflow of Genetic Information Extraction. (1) We extracted all sentences mentioning “BRCA1” and “BRCA2” gene from patients’ clinical notes using an NLP system, MedTagger. (2) We characterized the universality of extracted sentences using sf-ipf. The sf-ipf setting is similar to tf-idf commonly used in document-topic modeling. (3) We applied point-wise mutual information to automatically rank each word based on their inequality score and identified topic-indicating words. (4) We developed rules for automatic context inference in an iterative process by examining sentences with sf-ipf < 0.05 and a random sample of the rest of the sentences. (5) Manual evaluation was conducted by experts

Figure 1 illustrated the workflow of our NLP system. Step (1) is shared by both rule-based and machine learning systems while the rest applies for the rule-based system only. In step (1), we extracted all instances mentioning “*BRCA1”* and “*BRCA2”* gene from patients’ clinical notes using an NLP system MedTagger [34]. MedTagger enabled a series of NLP processes including dictionary-based concepts indexing, keyword mention lookup, and regular expression matching [35]. Duplicate sentences within each patient’s array were removed and only the earliest sentence was kept. We first applied regular expression to identify gene names that follow HUGO Gene Nomenclature [36] and variant names that follow Human Genome Variation Society (HGVS) Nomenclature [37]. We replaced gene names (except *BRCA1/2*) with “GENE”, mutation nomenclature with “MUTATION”. We then processed each sentence by removing stopword, punctuations, numbers and kept only Unified Medical Language System (UMLS) [38] identifiable concepts. We also normalized each word using the Stanford Core NLP tool [39] which includes sentence splitting, tokenization, Part-of-Speech tagging, lemmatization, negation detection and dependency parsing.

We characterized the universality of extracted sentences using *sf-ipf* (sentence frequency*-*inverse patient frequency). We assumed that sentences containing information regarding a patient’s genetic test results should be relatively unique and specific to patients’ results thus have low sf-ipf. The *sf-ipf* setting was similar to *tf-idf* (term frequency*-*inverse document frequency) [40] commonly used in document-topic modeling [41]. For sentence s,

The *sf-ipf* (s) = sf(s)*ipf(s) where:

1 $$sf\left( s \right) = \frac{{\# { }\;{\text{sentence}}\;{\text{ s }}}}{{{\text{total }}\# { }\;{\text{sentence }}}} ,\quad ipf\left( s \right) = \frac{{{\log}\left( {{\text{total }}\# \;{\text{ patients }}\left( {\text{N}} \right)} \right)}}{{\# \;{\text{ patients }}\;{\text{with }}\;{\text{sentence }}\;{\text{s}}}}$$

Sf-ipf sentence scores were calculated to represent how universal each sentence to actual genetic testing results. Two abstractors with medical education background reviewed sentences with sf-ipf < 0.5 (n = 46) plus additional randomly selected 50 sentences in the initial phase to determine topics. We chose 0.5 as a cut off for sf-ipf because it generates a managable size of initial senentece set for review. Stratified sampling from two different sentence pools enabled us to create a balanced initial training set for rule development.

## NLP system development and evaluation

### Rule-based system

As shown in Fig. 1, after the identification of topics and relevant topic-indicating words, we developed a rule-based system for automatic topic assignment in an iterative process. After the initial topic assignment, point-wise mutual information (PMI) [42, 43] was calculated and topic-indicating words were identified. Domain expertise was also applied to identify topic-indicating words. Rules were developed iteratively considering topic-indicating words and their proximity to genetic information mentions. If any sentence could not be categorized into existing topics, abstractors discussed with a domain expert to add new topics. We updated topic-indicating words and proximity rules iteratively until top PMI words and topic-indicating words converged. For sentences containing positivity indicators, we extracted both its pathogenic status (e.g. pathogenic, VUS, wildtype) and detailed variant information (if available) following the HGVS nomenclature. A heuristic rule was developed in which a positivity indicator was first assigned to the closet gene mention but was rejected subsequently if the gene mention and indicator were beyond five-word distance apart. In total, abstractors reviewed 300 randomly selected sentences in the development process (three iterations).

#### Machine learning system

We also developed a machine learning system to classify sentence topics utilizing 396 sentences evaluated during the iterative rule development process and were evaluated using ten-fold cross-validation. Input sentences were tokenized and were then transformed into vectors and fed into the Random Forest classifier. The reason we chose to use the Random Forest classifier is because of its good interpretability [44]. We developed two classifiers: one with the complete seven topics and another with only four topics: “Information”, “Positive”, “Negative”, and “VUS” where “Information”, “Evaluation”, “Order” and “Insurance” were combined into “Information”. The reason we introduced two classification tasks is to evaluate data (feature) quality of downstream analysis tasks with respect to different topic granularity. For example, to examine whether patient education (“Information”) can initiate more ordering of tests (“Order”), we will need to perform the seven-topic task. In another use case where we only wanted to extract patients’ genotype, a four-topic task would suffice. Therefore, in our study, we compared the performance differences between the two classification tasks.

#### Evaluation

Two abstractors manually assigned topics to another selected randomly 100 sentences according to final definitions of topics. Manual review results were compared to topic predictions generated by both rule-based and machine learning systems (two classifiers). Precision, recall, F-measure as well as an inter-rater agreement (Kappa statistics) were calculated using predictions from the finalized rules. Precision = $\frac{{{\text{True }}\;{\text{Positive}}}}{{{\text{True }}\;{\text{Positive}} + {\text{False }}\;{\text{Positive}}}}$, Recall = $\frac{{{\text{True}}\;{\text{ Positive}}}}{{{\text{True}}\;{\text{ Positive}} + {\text{False }}\;{\text{Negative}}}}$, F-measure = $\frac{{2{\text{*True }}\;{\text{Positive}}}}{{2{\text{*True }}\;{\text{Positive}} + {\text{False }}\;{\text{Positive}} + {\text{False }}\;{\text{Negative}}}}$.

### Temporal examination of topics and data quality examination

We visualized and examined the temporal pattern of topics regarding *BRCA1/2* by first ranking topic occurrence order in each patient’s timeline and then summarize the counts of ranks for different topics. Temporal distributions of topics were mapped into a “Topic” versus “Rank” heatmap. Counts were normalized and were presented in percentage (each row/topic sums up to 100%). Through the temporal visualization of each patient’s timeline, we were able to examine the order of types of encounters (“topics”) throughout a patient medical journey.

We examined the completeness of extracted genetic results in topics “Negative”, “Positive” or “VUS” by mapping them to an internal clinical genomic data model for genetic test result curation (Table 1). The data model was created by the integration of data elements from three different genetic testing report sources: Foundation Medicine, Tempus, and Mayo Clinic Internal Laboratory Service Line. We focused on examining the capture rate of (percentage of sentences with extracted genetic information that can be represented by) five data elements (bolded in Table 1): “Variant_Type”, “Variant_Source”, “Variant_Pathogeneity_Reported”, “Variant_Classification” and “HGVS_Short”. This data model has not been evaluated and was used just for data management purposes.

**Table 1:** Internal clinical genomics data model for genetic test result curation

Field_Name	Descriptions	Allowed_Values	Examples	Completeness
Hugo_Symbol	Gene Symbol	String	EGFR	**75% for Positive, 20.8% for VUS**
Ensemble_Gene_ID	Ensemble Gene ID	String starting with prefix "ENSG"	ENSG00000146648	0%
Transcript_ID	Transcript ID	String starting with prefix "NM" or "NR"	ENSG00000146648	0%
De_sample_ID	De-identified sample ID	String	MCM123	-
Pathogeneity_Report_Date	Date of initial genetic report	String of mm/dd/yyyy	2/12/2006	0%
**Variant_Type**	Type of variants	String of "SNP", "INDEL", “CNV”, “Rearrangement”	SNP	**50%**
**Variant_Source**	Somatic or germline	String of "somatic" or "germline"	germline	**26.4%**
**Variant_Pathogenicity**	Initial reported pathogenicity	String of "actionable", "pathogenic", or "VUS"	pathogenic	**43.8%**
**Variant_Classification**		**Selected strings from some of below:** Frame_Shift_Del, Frame_Shift_Ins, In_Frame_Del, In_Frame_Ins, Missense_Mutation, Nonsense_Mutation, Silent, Splice_Site, Translation_Start_Site, Nonstop_Mutation, 3′UTR, 3′Flank, 5′UTR, 5′Flank, IGR, Intron, RNA, Targeted_Region, De_novo_Start_InFrame, De_novo_Start_OutOfFrame	Missense_Mutation	**6.3%**
**HGVS_Short**	HGVS nomenclature for cDNA and Amino Acid Change	A string following HGVS nomenclature to detonate protein amino acid change	p.Arg149Trp	**25%**
NCBI_Build	The Genome Reference Consortium Build		"GRCh37"	0%
Chromosome	Chromosome of event	String of "1"-"22", "X", "Y", "M"	"7"	0%
Start_Position	Start position of event	Numerical		0%
End_Position	End position of event	Numerical		0%
Strand	Strand that the mutation is reported for	Character of "+" or "−"	"+"	0%
Variant_Allele_Freq	Percentage of variant presence in the sample	Numerical between 0 and 100	30	0%
BP_Coverage	Base-pair coverage	Numerical	270	0%
Variant_Pathogenicity_Updated	Updated pathogenicity			-
Pathogeneity_Update_Date	Update date			-

### Association analaysis between mutation and targeted therapy

We expanded our analysis by examining whether *BRCA1/2* mutation information from Foundation reports resulted in discussion or prescription of PARP inhibitors. We classified patients’ genetic mutation status using validated results from Foundation Medicine reports: BRCA Mutated, BRCA VUS, and BRCA Negative. We extracted PARP discussion status by identifying “Information” sentences that also cover information of PARP inhibitors. PARP prescription status was retrieved by extracting patients’ prescription history from Mayo Clinic Unified Data Platform (UDP) [45, 46] structured Medication table, clinical data management (CDM) reports which were used to record clinical trial drug administration history, and semi-structured clinical notes “Current Medication” section. We searched for entries with “Olaparib”, “Rucaparib”, and “Niraparib” in UDP and CDM reports and terms “PARP”, “PARPi”, “Olaparib”, “Rucaparib”, “Niraparib” as well as their brand names (“Lyparza”, “Rubraca”, “Zejula”) in semi-structured clinical notes. Association analysis was performed between (1) BRCA mutation status versus PARP discussion status and (2) BRCA mutation status versus PARP prescription status.

## Result

We examined the corpus using an NLP-based approach and identified seven topics regarding *BRCA1/2* (“Topic identification to address contextual variability in unstructured clinical notes” section). The performance of the rule-based and machine learning-based NLP systems were evaluated and compared (“NLP system for automatic topic classification” section). A timeline view of patients’ medical journey regarding *BRCA1/2* was visualized and temporal distributions of topics demonstrated a representative pattern for Precision Medicine practice (“Temporal visualization and examination of topics” section). Data quality examination revealed incompleteness and discrepancy regarding the capture of genetic information in unstructured clinical notes thus combining different sources is necessary (“BRCA1/2-related real-world data quality in unstructured clinical notes” section). Finally, using cleaned RWD, we were able to identify a significant association between *BRCA1/2* mutation and the prescription of PARP inhibitors (“Mutation-medication association” section).

### Topic identification to address contextual variability in unstructured clinical notes

In total, we extracted 1179 sentences that contain keywords *BRCA1*, *BRCA2* for 122 patients while the remaining 74 of 196 patients in the cohort cannot find any matching records with these keywords. After removing duplicate sentences, there were a total of 682 unique sentences. Additional file 1: Table S1 listed top 10 lowest sf-ipf sentences.

After reviewing sentences with sf-ipf < 0.5 (n = 46) plus 50 additional randomly selected sentences in the initial phase, two abstractors and one domain expert classified the initial sentence topics into seven topics: Information, Evaluation, Insurance, Order, Negative (Mutation), Positive (Mutation), and VUS. Three of them (Negative, Positive, VUS) were related to patient genetic information. The scope of each topic was also defined clearly to enable evaluation by abstractors (Table 2). In the rule development process, abstractors identified no sentence that cannot be classified into seven topics.

**Table 2:** Definition of topics for sentences

Topic	Definition
Information	General information about guideline, genetic testing panels, pathways and biological implications, etc
Evaluation	Physician estimating risk of having BRCA1/2 mutation, benefit of taking genetic test, etc. & Record any BRCA1/2 muation-carrier relative and risk of being a BRCA1/2 carrier from family history
Insurance	Concerns over insurance coverage
Order	Physician recording ordering of tests or waiting for results
Negative (mutation)	Negative mutation result from genetic test
Positive (mutation)	Positive mutation found from genetic test
VUS	VUS found from genetic test

### NLP system for automatic topic classification

**Fig. 2:**
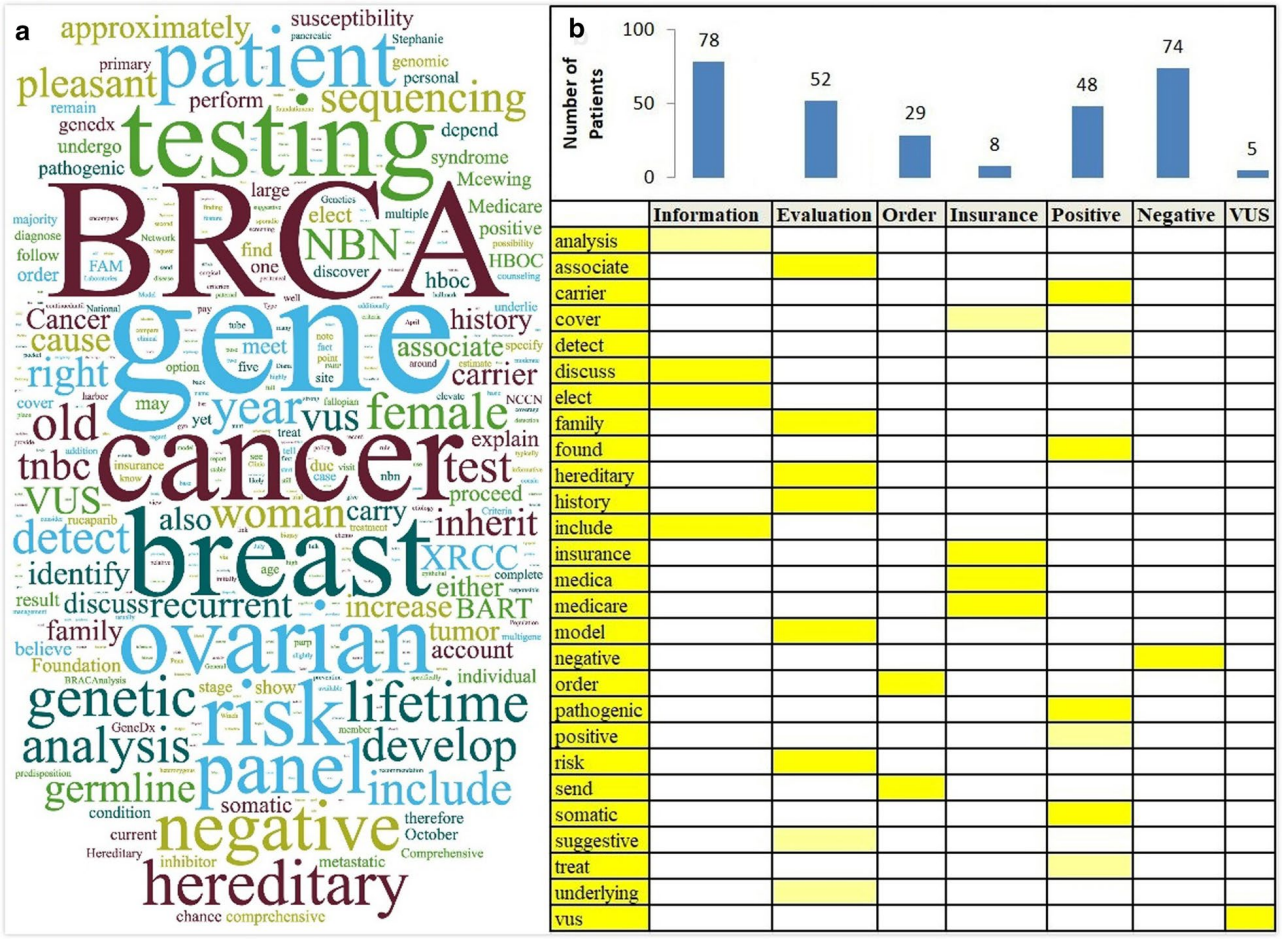
**a** Word-cloud based on non-duplicate sentences extracted from EHRs regarding BRCA1/2; **b** Heatmap of Top PMI (Inequality score) Words in Different Topics (Yellow = Indicating Words; Light Yellow = Conditional Indicating Words)

Identification of topic indicating words and assigning topics to each sentence using regular expression involved an iterative evaluation process until top PMI words and topic indicating words converged. According to 682 BRCAness-related unique sentences in EHRs, a word-cloud was generated as Fig. 2a, showing the sizes of words proportional to their frequencies in the corpus. Despite that some frequent words were implicitly related to BRCAness, such as “breast” “ovarian” “cancer” and “family” “history”, there lacked clinical contexts to cluster words that share a similar “topic”. After utilizing rule-based topic classification, specific words could be assigned to different topics related to either type of medical encounters (e.g. Information, Evaluation) or genetic results (Positive/Negative/VUS). Shown as Fig. 2b, a highly sparse pattern of the word-topic matrix indicated the specificity of representative words, e.g. “family”, “history” and “hereditary” were exclusive to the topic of “Evaluation”.

The final evaluation of our system was conducted by two abstractors with an inter-rater agreement (Kappa statistics) of 0.95, and achieved an overall precision of 0.87, recall of 0.93, and F-measure of 0.91. Performance metrics of machine learning classifiers were listed in Tables 3 and 4. We could see that four-topic achieved better overall performance. Performance for classifying “Order” and “Insurance” was not ideal. This may be due to limited instances for these two topics (number of patients with “Order” topic = 29 and the number of patients with “Insurance” topic = 8).

**Table 3:** Performance of machine learning system on four-topic classification

Class	Precision	Recall	F-measure
Information	0.933	0.925	0.929
Positive	0.757	0.8	0.778
Negative	0.879	0.879	0.879
VUS	1	0.958	0.979
Overall	0.901	0.899	0.9

**Table 4:** Performance of machine learning system on seven-topic classification

Class	Precision	Recall	F-Measure
Information	0.714	0.645	0.678
Evaluation	0.911	0.895	0.903
Insurance	1	0.625	0.769
Order	1	0.5	0.667
Positive	0.707	0.829	0.763
Negative	0.789	0.909	0.845
VUS	0.92	0.958	0.939
Overall—ML	0.833	0.823	0.82
Overall—rule	0.87	0.93	0.91

Feature importance of two random forest classifiers was calculated based on Gini impurity/information gain [47] and was provided in Additional file 2: Table S2 and Additional file 3: Table S3. Feature importance from random forest classifiers agreed with our rule-based PMI calculation in a majority of cases. For example, “vus”, “negative”, “pathogenic”, “order”, “risk” and “analysis” were considered important topic-indicators and they were ranked high in both “average impurity decrease” and “number of nodes using that attribute”.

### Temporal visualization and examination of topics

**Fig. 3:**
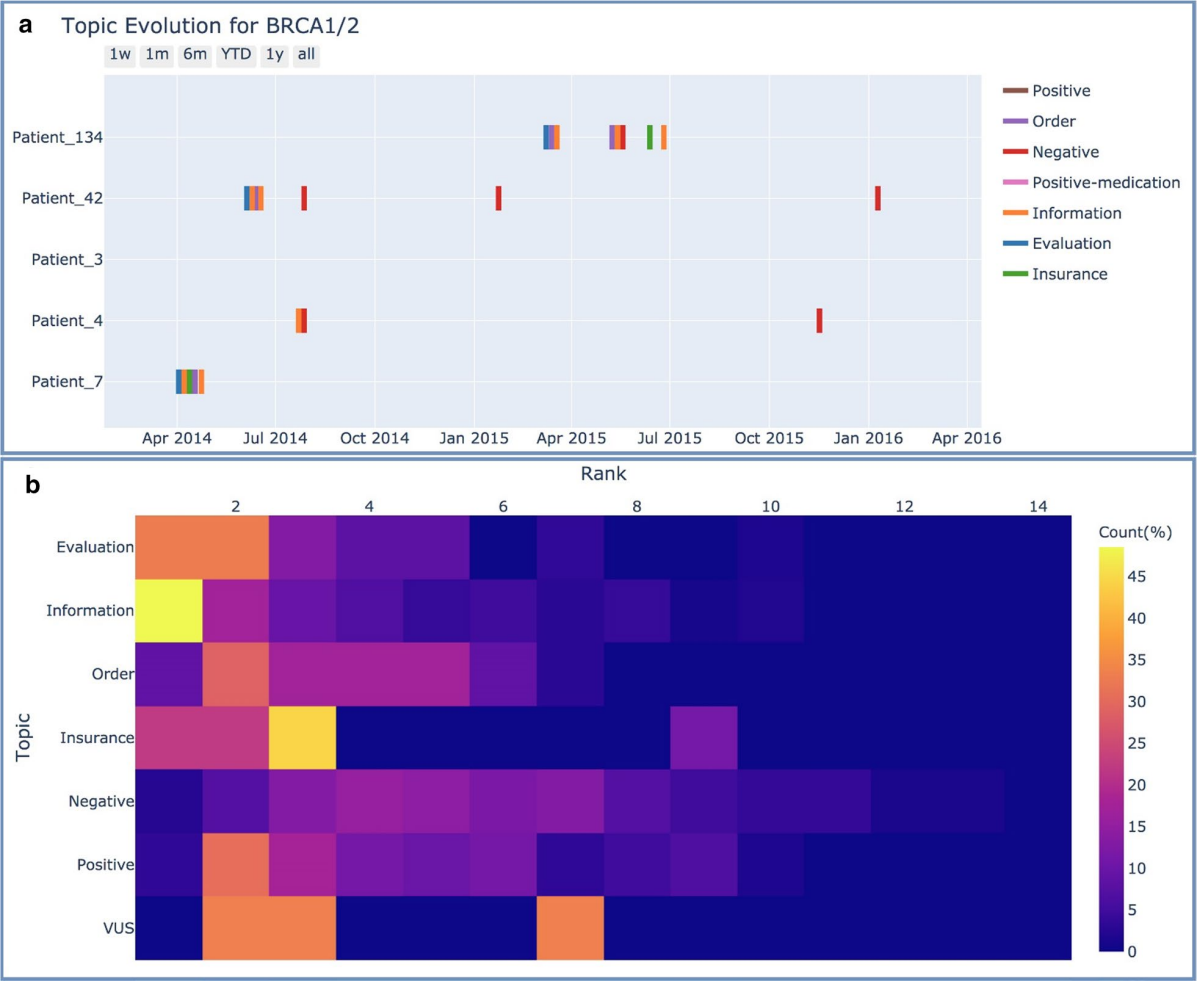
**a** Timeline view of Individual Patients’ Medical Journey; **b** Heatmap of A Summarized View for Temporal Pattern of Topics Throughout Patients’ Medical Journey. Y-axis = Topics, X-axis = Ranks of Occurrence Order in Patient Timelines. Counts were Normalized and were Presented in Percentage (each row/topic sums up to 100%)

In order to examine temporal patterns of topic distributions, *BRAC1/2* related events were mapped to a timeline. Figure 3a listed individual timelines for 5 patients. We could see that patients share a similar temporal pattern of the medical journey starting from “Evaluation”, “Information”, followed by “Order”, and optionally “Insurance” and finally genetic information (“Positive”, “Negative”, and “VUS”). Figure 3b demonstrated a summarized count percentage heatmap of the temporal order of topics (1 as earliest encounter, 14 as the last encounter, total numbers of encounters for each patient varies) for all the patients. The results from Fig. 3b agreed with our observation from the individualized view in Fig. 3a that “Evaluation” and “Information” encounters often appear earliest in the timeline. The initial topic of “Order” of genetic tests followed immediately after “Evaluation” was performed and “Information” was communicated with patients. “Insurance” occurred more frequently after the initial proposal of “Order” and would sometimes take several encounters to receive confirmation from insurance companies and proceeded with genetic tests. Result-related topics (“Positive”, “Negative”, and “VUS”) were mentioned repeatedly because every encounter, physicians will refer back to medical history to initiate/change treatment plans. Among all result-related topics, “Positive” and “VUS” results were reported and documented earlier than “Negative” results.

### BRCA1/2-related real-world data quality in unstructured clinical notes

We examined the data quality of RWD from unstructured sources with a focus on completeness and discrepancies of genetic test results. We compared the results documented in unstructured clinical notes EHR records versus Foundation Medicine reports. Among Foundation positive patients (N = 12), 75% of patients (N = 9) had matching EHR records of their positive *BRCA1/2* mutation. For VUS, missingness was much larger – only 5 patients out of 24 (20.8%) have their VUS recorded in the EHR. From Fig. 2b, we could see that we have more positive cases from EHR records (N = 48) than Foundation-provided positive patients (N = 9). The reason for this is our information extraction system extracted previous germline *BRCA1/2* panel test results as well.

Among all data elements in Table 1, five data elements “Variant_Type”, “Variant_Source”, “Variant_Pathogeneity_Reported”, and “Variant_Classification” were considered most relevant to represent genetic information. Completeness of genetic information captured in clinical notes was listed in the last column of Table 1. We found that the current capture rate of all data elements was low: “Variant_Type” (8 out of 16, 50%), “Variant_Pathogeneity_Reported” (7 out of 16, 43.8%) and “Variant_Classification” (1 out of 16, 6.3%), HGVS_short (4 out of 16, 25%) should be recorded for both positive and VUS patients while “Variant_Source” (24 out of 91, 26.4%) should be available for all negative, positive and VUS patients because it can be derived from genetic testing panel type. Among four data elements, “Variant_Classification” was least frequently documented—only one patient with a “splice_site” variant was documented. In some cases, “Variant_Type” was not extracted explicitly but can be inferred from extracted information that follows HGVS nomenclature, for example, S34F and c.7759C > T is a “single-nucleotide variant” and amplification is a “copy number variation”. Among five data elements, “Variant_Classification” was least frequently documented—only one patient with a “splice_site” variant was documented.

**Fig. 4:**
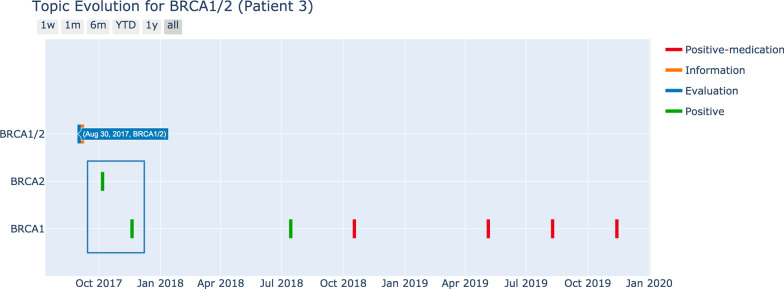
Timeline view of medical journey of patient #3 revealed discrepancy in clinical notes documentation

Figure 4 displayed a timeline view of a single patient (Patient 3). In this view, we could see that this patient had a discussion with the physician about the risk of having *BRCA1/2* and the benefits of having the genetic test on 08/30/2017. After the discussion, the patient took the test, and the results returned positive. But there was a discrepancy in clinical notes documentation where it first documents the results as a positive mutation in *BRCA2* but later revised it to *BRCA1* (rectangled). This example demonstrated the benefit of using a timeline view to perform data quality checks in the future.

### Mutation-medication association

**Fig. 5:**
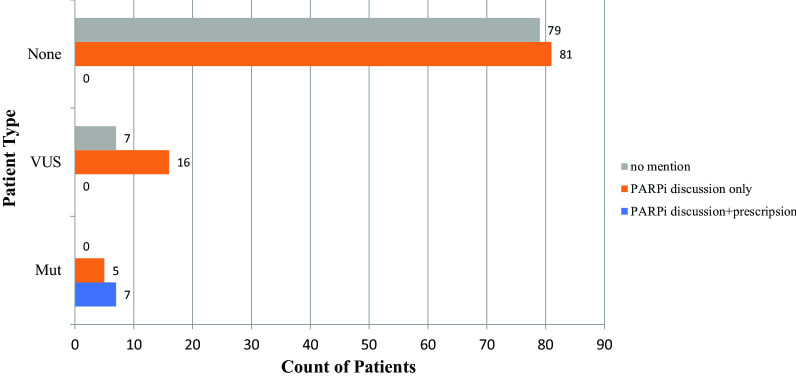
Patient BRCA1/2 mutation status versus PARP inhibitor (“Olaparib”, “Rucaparib”, and “Niraparib”) Prescription. Mut = with BRCA1/2 mutation, VUS = with BRCA1/2 VUS, None = none of the above. “PARPi discussion only” = patients with only discussion or recorded activity related to this drug; “PARPi discussion + prescription” = patients with discussion as well as confirmed prescriptions from UDP, CDM reports and clinical notes “current_medication” section

Figure 5 showed patient’s genetic mutation status validated by Foundation Medicine reports, patients’ PARP inhibitor discussions status from clinical notes, and patients’ PARP inhibitor prescription status from UDP, CDM reports, and clinical notes combined. “PARPi discussion” patient type referred to patients with the only discussion related to this drug while “PARPi prescription” referred to patients with confirmed prescriptions from UDP, CDM reports, and clinical notes “current_medication” section. “PARPi discussion + prescription” referred to patients with both discussions related to this drug in clinical notes and confirmed prescriptions. Because there was no patient that had “PARPi prescription” without “PARPi discussion”, we didn’t display “PARPi prescription” in the figure. Fisher's Exact Test was used to test differences between patients with different *BRCA1/2* mutation status. Results from the test on the count data revealed a strong association between the patient’s genetic mutation in *BRCA1/2* and the prescription of PARP inhibitors with *p*-value = 0.0004.

### Discussion

As the use of gene mutation data (somatic and germline) has become a common practice across all subspecialties of oncology for the purpose of therapeutic planning, RWD that reflected utilization and outcomes of such testing was buried within clinical notes. Contextual variability introduced challenges for data extraction. Therefore, identifying topics that revealed clinical contexts before applying the NLP system can facilitate accurate extraction of genetic information from unstructured clinical notes. In addition, we proposed a machine learning system to classify extracted information into different “topics”.

We demonstrated that proposed “topics” and their distributions represented patients’ medical journeys and clinical decision-making process. We also examined the data quality of RWD and identified issues such as incompleteness and discrepancies. Finally, further association analysis enhanced our understanding of the utility of genetic information in treatment selections.

Our genetic information extraction system revealed discrepancies and missingness of genetic data in EHRs: (1) *BRCA1/2* mutation information was captured 75% and VUS only 20.8% (2) certain data fields (e.g. variant type or source) within clinical notes have a low rate of capture. Although we considered some of these fields to be important, such as VUS as well as detailed variant information, the low rate of documentation indicates that such data are not essential in the routine management of patients. Indeed, oncology providers only need to know whether a specific gene has a pathogenic mutation to determine eligibility for FDA-approved targeted agents; the specific variant details do not contribute to clinical decision making.

Although the missingness of data, in this case, is based on contemporary needs, the evolving knowledge in the field of clinical genomics could lead to a reclassification of a VUS to pathogenic within a few years. If we are able to automatically populate unstructured genetic information into a federated structured data warehouse, we will probably benefit from a more detailed record of variant information by developing clinical decision support tools or EHR plug-in that consistently updates variant interpretations and informs physicians in a timely manner. Looking beyond the scope and benefit of a single institution, adoption of a standardized data model to store clinical genomics information (variant + clinical phenotypes) in a structured database could benefit clinical practice in two ways: 1) reducing dependency on clinician’s documentation to capture genetic information given existing variability in documentation styles, especially when genetic tests are from an outside vendor and data capture rate in EHR is low [24]; 2) enabling multi-institutional collaboration for variant curation: examples for such collaborative effort are TCGA (The Cancer Genome Atlas) [48] and AACR Project GENIE (Genomics Evidence Neoplasia. Information Exchange) [49] which focused on generating evidence for precision medicine by integrating cancer genomic data with clinical outcome data.

Our association analysis was performed on “BRCAness” where we evaluated *BRCA1/2* mutation status versus the prescription of PARP inhibitors. Also, given that *BRCA1/2* is a strong indicator for the prescription of PARP inhibitors, discussion of PARP inhibitors happens in over 50% of the patients in this cohort. The results agreed with our previous knowledge that BRCAness [5, 50], which describes somatic *BRCA1/2* homologous recombination repair (HRR) defect with no detectable germline *BRCA1/2* mutation, provide actionable information in clinical decision-making.

The limitation of our work is that we only evaluated the extraction of *BRCA1/2* related information and the topics we identified in *BRCA1/2* related information may not apply to other use cases. The generalizability of our methods needs further evaluation. Another limitation of our work is that we only tested the performance of random forest classifier when comparing between machine learning and rule-based systems.

Other tree-based ensemble models such as XGBoost [51] might achieve superior performance than random forest and it also provides feature importance. However, feature importance orderings are very different for each of the three options (weight, cover, gain) provided by XGBoost, which might need additional evaluation before a conclusion can be drawn. Therefore, this might be considered as a future attempt but is beyond the scope of this manuscript. In future work, we will apply this system to extract other HRR genes as they were also shown to cause cellular sensitivity to PARP inhibitors [52]. We will analyze topic distributions of those genes when mentioned in clinical notes to confirm/prioritize potentially actionable genes. Secondly, we can also use topics defined in this work to examine the benefit of adequate patient education (“Information” topics) in promoting shared decision-making and precision medicine outcomes. Finally, we will expand our method on other cohorts including patients with Tempus or Mayo in-house genetic tests or external cohorts from other institutions to test the generalizability of our method. After we have constructed a more comprehensive cohort, we will be able to examine the clinical utility (measured in overall survival or likelihood of being matched to a clinical trial or a targeted therapy) of genetic testing panels of different sizes (single gene vs multi-gene panel).

## Conclusions

In our work, we demonstrated a framework to generate RWE using RWD from different clinical sources: (1) Applying the NLP system that can resolve contextual variability to extract RWD from unstructured clinical notes. (2) Examination of data quality issues such as incompleteness and discrepancies in retrieved data. (3) Due to limited data quality, manual data cleaning is needed before further analysis can be performed. (4) Using cleaned RWD to generate RWE. From our use case, we found that currently, the rule-based NLP system achieved the best performance. Data quality issues such as incompleteness and discrepancies exist and vary by data type. Finally, we were able to use cleaned RWD to show that the real-world association of *BRCA1/2* and discussion/prescription of PARP inhibitor is significant.

## Supplementary Information

**Additional file 1: Table S1**. Top 10 Most Universal (Lowest sf-ipf) Sentences.

**Additional file 2: Table S2**. Feature Importance of Random Forest Classifier for Classification of Four Topics (Information, Positive, Negative, VUS).

**Additional file 3: Table S3**. Feature Importance of Random Forest Classifier for Classification of Seven Topics (Information, Evaluation, Order, Insurance, Positive, Negative, VUS).

## Data Availability

The data used in this study cannot be shared because of the patient health information included in the texts.

## Abbreviations

RWD: Real-world data
RWE: Real-world evidence
NLP: Natural language processing
VUS: Variants of unknown significance
EHR: Electronic health record
sf-ipf: Sentence frequency-inverse patient frequency
tf-idf: Term frequency-inverse document frequency
HGVS: Human Genome Variation Society
PMI: Point-wise mutual information
UMLS: Unified Medical Language System
PARP: Poly-adenosyldiphosphate-ribose polymerase
UDP: Unified Data Platform
CDM: Clinical data management
HRR: Homologous recombination repair
GENIE: Genomics Evidence Neoplasia Information Exchange
TCGA: The Cancer Genome Atlas

## References

[1] Couch FJ, Nathanson KL, Offit K (2014). Two decades after BRCA: setting paradigms in personalized cancer care and prevention. Science.

[2] Pruthi S, Gostout BS, Lindor NM. Identification and management of women with BRCA mutations or hereditary predisposition for breast and ovarian cancer. In: Mayo Clinic proceedings (Elsevier); 2010. p. 1111–20. 10.4065/mcp.2010.0414 PMC2996153 21123638

[3] Venkitaraman AR (2014). Cancer suppression by the chromosome custodians, BRCA1 and BRCA2. Science.

[4] Rios J, Puhalla S (2011). PARP inhibitors in breast cancer: BRCA and beyond. Breast Cancer.

[5] Turner N, Tutt A, Ashworth A (2004). Hallmarks of'BRCAness' in sporadic cancers. Nat Rev Cancer.

[6] Krynetskiy E, McDonnell P (2007). Building individualized medicine: prevention of adverse reactions to warfarin therapy. J Pharmacol Exp Ther.

[7] Evans WE, Relling MV (2004). Moving towards individualized medicine with pharmacogenomics. Nature.

[8] Trusheim MR, Berndt ER, Douglas FL (2007). Stratified medicine: strategic and economic implications of combining drugs and clinical biomarkers. Nat Rev Drug Discovery.

[9] Chantrill LA, Nagrial AM, Watson C, Johns AL, Martyn-Smith M, Simpson S, Mead S, Jones MD, Samra JS, Gill AJ (2015). Precision medicine for advanced pancreas cancer: the individualized molecular pancreatic cancer therapy (IMPaCT) trial. Clin Cancer Res.

[10] Green MJ, Botkin JR (2003). Genetic exceptionalism in medicine: clarifying the differences between genetic and nongenetic tests. Ann Intern Med.

[11] Holtzman NA, Murphy PD, Watson MS, Barr PA (1997). Predictive genetic testing: from basic research to clinical practice. Science.

[12] Relling MV, Evans WE (2015). Pharmacogenomics in the clinic. Nature.

[13] Reyna VF, Lloyd FJ, Whalen P (2001). Genetic testing and medical decision making. Arch Intern Med.

[14] Lerman C, Narod S, Schulman K, Hughes C, Gomez-Caminero A, Bonney G, Gold K, Trock B, Main D, Lynch J (1996). BRCA1 testing in families with hereditary breast-ovarian cancer: a prospective study of patient decision making and outcomes. JAMA.

[15] Kurian AW, Li Y, Hamilton AS, Ward KC, Hawley ST, Morrow M, McLeod MC, Jagsi R, Katz SJ (2017). Gaps in incorporating germline genetic testing into treatment decision-making for early-stage breast cancer. J Clin Oncol.

[16] Weiskopf NG, Weng C (2013). Methods and dimensions of electronic health record data quality assessment: enabling reuse for clinical research. J Am Med Inform Assoc.

[17] Banda JM, Callahan A, Winnenburg R, Strasberg HR, Cami A, Reis BY, Vilar S, Hripcsak G, Dumontier M, Shah NH (2016). Feasibility of prioritizing drug–drug-event associations found in electronic health records. Drug Saf.

[18] De Moor G, Sundgren M, Kalra D, Schmidt A, Dugas M, Claerhout B, Karakoyun T, Ohmann C, Lastic P-Y, Ammour N (2015). Using electronic health records for clinical research: the case of the EHR4CR project. J Biomed Inform.

[19] Hripcsak G, Albers DJ (2013). Next-generation phenotyping of electronic health records. J Am Med Inform Assoc.

[20] Sherman RE, Anderson SA, Dal Pan GJ, Gray GW, Gross T, Hunter NL, LaVange L, Marinac-Dabic D, Marks PW, Robb MA (2016). Real-world evidence—what is it and what can it tell us. N Engl J Med.

[21] Klonoff DC (2019). The expanding role of real-world evidence trials in health care decision making. J Diabetes Sci Technol.

[22] Khozin S, Blumenthal GM, Pazdur R (2017). Real-world data for clinical evidence generation in oncology. JNCI J Natl Cancer Inst.

[23] Yadav P, Steinbach M, Kumar V, Simon G (2018). Mining electronic health records (EHRs): a survey. ACM Comput Surv CSUR.

[24] Zhao Y, Yu H, Fu S, Shen F, Davila JI, Liu H, Wang C (2020). Data-driven sublanguage analysis for cancer genomics knowledge modeling: applications in mining oncological genetics information from patient’s genetic reports. AMIA Summits Transl Sci Proc.

[25] Wei W-Q, Denny JC (2015). Extracting research-quality phenotypes from electronic health records to support precision medicine. Genome Med.

[26] Wang G, Jung K, Winnenburg R, Shah NH (2015). A method for systematic discovery of adverse drug events from clinical notes. J Am Med Inform Assoc.

[27] Liu S, Wang L, Ihrke D, Chaudhary V, Tao C, Weng C, Liu H (2017). Correlating lab test results in clinical notes with structured lab data: a case study in hba1c and glucose. AMIA Summits Transl Sci Proc.

[28] Lee KH, Kim HJ, Kim Y-J, Kim JH, Song EY (2020). Extracting structured genotype information from free-text HLA reports using a rule-based approach. J Korean Med Sci.

[29] Son JH, Xie G, Yuan C, Ena L, Li Z, Goldstein A, Huang L, Wang L, Shen F, Liu H (2018). Deep phenotyping on electronic health records facilitates genetic diagnosis by clinical exomes. Am J Hum Genet.

[30] Van Driest SL, Wells QS, Stallings S, Bush WS, Gordon A, Nickerson DA, Kim JH, Crosslin DR, Jarvik GP, Carrell DS (2016). Association of arrhythmia-related genetic variants with phenotypes documented in electronic medical records. JAMA.

[31] Chan KR, Lou X, Karaletsos T, Crosbie C, Gardos S, Artz D, Rätsch G. An empirical analysis of topic modeling for mining cancer clinical notes. In: 2013 IEEE 13th international conference on data mining workshops (IEEE); 2013. p. 56–63.

[32] Shirts BH, Salama JS, Aronson SJ, Chung WK, Gray SW, Hindorff LA, Jarvik GP, Plon SE, Stoffel EM, Tarczy-Hornoch PZ (2015). CSER and eMERGE: current and potential state of the display of genetic information in the electronic health record. J Am Med Inform Assoc.

[33] Guan M, Cho S, Petro R, Zhang W, Pasche B, Topaloglu U (2019). Natural language processing and recurrent network models for identifying genomic mutation-associated cancer treatment change from patient progress notes. JAMIA Open.

[34] Liu H, Bielinski SJ, Sohn S, Murphy S, Wagholikar KB, Jonnalagadda SR, Ravikumar K, Wu ST, Kullo IJ, Chute CG (2013). An information extraction framework for cohort identification using electronic health records. AMIA Summits Transl Sci Proc.

[35] Torii M, Wagholikar K, Liu H (2011). Using machine learning for concept extraction on clinical documents from multiple data sources. J Am Med Inform Assoc.

[36] Eyre TA, Ducluzeau F, Sneddon TP, Povey S, Bruford EA, Lush MJ (2006). The HUGO gene nomenclature database, 2006 updates. Nucleic Acids Res.

[37] den Dunnen JT, Dalgleish R, Maglott DR, Hart RK, Greenblatt MS, McGowan-Jordan J, Roux AF, Smith T, Antonarakis SE, Taschner PE (2016). HGVS recommendations for the description of sequence variants: 2016 update. Hum Mutat.

[38] Bodenreider O (2004). The unified medical language system (UMLS): integrating biomedical terminology. Nucleic Acids Res.

[39] Manning CD, Surdeanu M, Bauer J, Finkel JR, Bethard S, McClosky D. The Stanford CoreNLP natural language processing toolkit. In: Proceedings of 52nd annual meeting of the association for computational linguistics: system demonstrations; 2014. p. 55–60.

[40] Jones KS (1972). A statistical interpretation of term specificity and its application in retrieval. J Doc.

[41] Bun KK, Ishizuka M. Emerging topic tracking system. In: Proceedings third international workshop on advanced issues of e-commerce and web-based information systems WECWIS 2001 (IEEE); 2001. p. 2–11.

[42] Church KW, Hanks P (1990). Word association norms, mutual information, and lexicography. Comput Ling.

[43] Cover TM, Thomas JA (2012). Elements of information theory.

[44] Du M, Liu N, Hu X (2019). Techniques for interpretable machine learning. Commun ACM.

[45] Horton I, Lin Y, Reed G, Wiepert M, Hart S (2017). Empowering Mayo Clinic individualized medicine with genomic data warehousing. J Personal Med.

[46] Kaggal VC, Elayavilli RK, Mehrabi S, Pankratz JJ, Sohn S, Wang Y, Li D, Rastegar MM, Murphy SP, Ross JL (2016). Toward a learning health-care system-knowledge delivery at the point of care empowered by big data and NLP. Biomed Inf Insights.

[47] Louppe G, Wehenkel L, Sutera A, Geurts P. Understanding variable importances in forests of randomized trees. In: Advances in neural information processing systems; 2013. p. 431–9.

[48] Tomczak K, Czerwińska P, Wiznerowicz M (2015). The cancer genome atlas (TCGA): an immeasurable source of knowledge. Contemp Oncol.

[49] Consortium APG (2017). AACR project GENIE: powering precision medicine through an international consortium. Cancer Discov.

[50] Byrum AK, Vindigni A, Mosammaparast N (2019). Defining and modulating ‘BRCAness’. Trends Cell Biol.

[51] Chen T, He T, Benesty M, Khotilovich V, Tang Y. Xgboost: extreme gradient boosting. R package version 04-2 2015:1–4.

[52] McCabe N, Turner NC, Lord CJ, Kluzek K, Białkowska A, Swift S, Giavara S, O'Connor MJ, Tutt AN, Zdzienicka MZ (2006). Deficiency in the repair of DNA damage by homologous recombination and sensitivity to poly (ADP-ribose) polymerase inhibition. Can Res.

