# Supplementary material for: Generating real-world evidence from unstructured clinical notes to examine clinical utility of genetic tests: use case in BRCAness
Source: BMC Med Inform Decis Mak. 2021 Jan 6;21:3. doi: 10.1186/s12911-020-01364-y (PMC7789545; doi:10.1186/s12911-020-01364-y)
Supplement: Supplementary file 1 — Additional file 1: Table S1. Top 10 Most Universal (Lowest sf-ipf) Sentences. [file 12911_2020_1364_MOESM1_ESM.docx]

Table S1 Top 10 Most Universal (Lowest sf-ipf) Sentences

| KEYWORD CONTAINING SENTENCE | sfipf | Topic |
| --- | --- | --- |
| Approximately 5-10% of breast cancer and 10-25% of ovarian cancer is hereditary and is usually due to mutations in BRCA1 and BRCA2 genes. | 0.01 | Discussion |
| Individuals who carry a mutation in BRCA1 or BRCA2 have an inherited susceptibility to develop cancer. | 0.01 | Discussion |
| We explained that BRCA1 and BRCA2 gene mutations account for 84% of hereditary breast and ovarian cancer (HBOC) and that the remaining 16% is caused by other genes that we have not discovered yet and other hereditary conditions. | 0.01 | Discussion |
| Males and females with BRCA1/BRCA2 mutations also face a slightly increased risk for pancreatic cancer. | 0.03 | Discussion |
| There is also an increased risk for prostate and breast cancer among males with a BRCA1/BRCA2 mutation. | 0.03 | Discussion |
| Germline BRCA1/2 wild-type. | 0.03 | Negative Mutation |
| The lifetime risk for breast cancer in women with a BRCA1/BRCA2 mutation is approximately 50%-87% and the lifetime risk for ovarian cancer is 15%-44%, depending on the specified gene. | 0.03 | Discussion |
| Mutations in either BRCA1 or BRCA2 cause Hereditary Breast and Ovarian Cancer syndrome (HBOC). | 0.04 | Discussion |
| BRCA1/BRCA2 testing includes both gene sequencing and deletion/duplication analysis. | 0.05 | Discussion |
| We discussed BRCA1 and BRCA2, two genes that are responsible for the majority of hereditary breast and ovarian cancer. | 0.05 | Discussion |
