# Supplementary material for: Generating real-world evidence from unstructured clinical notes to examine clinical utility of genetic tests: use case in BRCAness
Source: BMC Med Inform Decis Mak. 2021 Jan 6;21:3. doi: 10.1186/s12911-020-01364-y (PMC7789545; doi:10.1186/s12911-020-01364-y)
Supplement: Supplementary file 2 — Additional file 2: Table S2. Feature Importance of Random Forest Classifier for Classification of Four Topics (Information, Positive, Negative, VUS). [file 12911_2020_1364_MOESM2_ESM.docx]

Table S2. Feature Importance of Random Forest Classifier for Classification of Four Topics (Information, Positive, Negative, VUS)

| word | Attribute importance based on average impurity decrease | number of nodes using that attribute |
| --- | --- | --- |
| instance | 0.87 | 2 |
| way | 0.76 | 1 |
| vus | 0.7 | 128 |
| hx | 0.65 | 5 |
| request | 0.65 | 7 |
| negative | 0.58 | 135 |
| cari | 0.55 | 7 |
| rare | 0.55 | 8 |
| profile | 0.54 | 11 |
| no | 0.52 | 87 |
| order | 0.51 | 5 |
| issue | 0.51 | 2 |
| dakota | 0.51 | 2 |
| prostate | 0.51 | 2 |
| mut | 0.49 | 137 |
| hallmark | 0.49 | 2 |
| dna | 0.48 | 14 |
| carcinoma | 0.48 | 6 |
| specifically | 0.47 | 4 |
| number | 0.46 | 4 |
| er | 0.45 | 9 |
| along | 0.45 | 18 |
| trial | 0.45 | 8 |
| view | 0.44 | 7 |
| occur | 0.44 | 3 |
| tested | 0.42 | 4 |
| basic | 0.41 | 3 |
| myriad | 0.41 | 51 |
| pr | 0.4 | 11 |
| may | 0.4 | 24 |
| cost | 0.39 | 5 |
| right | 0.39 | 19 |
| link | 0.38 | 10 |
| diana | 0.38 | 10 |
| chemo | 0.38 | 11 |
| record | 0.38 | 11 |
| harness | 0.38 | 9 |
| not | 0.37 | 46 |
| see | 0.37 | 7 |
| know | 0.37 | 17 |
| tnbc | 0.37 | 31 |
| tend | 0.37 | 8 |
| accord | 0.37 | 16 |
| testing | 0.37 | 104 |
| addition | 0.37 | 21 |
| reassure | 0.37 | 6 |
| potential | 0.37 | 5 |
| cancer | 0.36 | 93 |
| gene | 0.35 | 117 |
| pass | 0.35 | 6 |
| small | 0.35 | 6 |
| repair | 0.35 | 12 |
| comprehensive | 0.35 | 27 |
| as | 0.34 | 26 |
| rule | 0.34 | 15 |
| panel | 0.34 | 57 |
| follow | 0.34 | 14 |
| within | 0.34 | 22 |
| suggestive | 0.34 | 8 |
| consultation | 0.34 | 8 |
| aunt | 0.33 | 8 |
| medicine | 0.33 | 12 |
| pleasant | 0.33 | 23 |
| complete | 0.33 | 30 |
| sequencing | 0.33 | 39 |
| come | 0.31 | 10 |
| send | 0.3 | 18 |
| bart | 0.3 | 45 |
| penn | 0.3 | 6 |
| test | 0.3 | 69 |
| reduce | 0.3 | 2 |
| stable | 0.3 | 8 |
| lovely | 0.3 | 5 |
| upwards | 0.3 | 6 |
| initially | 0.3 | 10 |
| likelihood | 0.3 | 11 |
| inheritance | 0.3 | 4 |
| fall | 0.29 | 7 |
| detect | 0.29 | 50 |
| though | 0.29 | 3 |
| medical | 0.29 | 4 |
| analysis | 0.29 | 81 |
| identify | 0.29 | 73 |
| positive | 0.29 | 30 |
| germline | 0.29 | 81 |
| fallopian | 0.29 | 9 |
| foundation | 0.29 | 38 |
| or | 0.28 | 43 |
| sample | 0.28 | 9 |
| method | 0.28 | 15 |
| arrange | 0.28 | 8 |
| risk | 0.27 | 71 |
| close | 0.27 | 5 |
| epcam | 0.27 | 46 |
| inform | 0.27 | 5 |
| proceed | 0.27 | 21 |
| recently | 0.27 | 9 |
| explanation | 0.27 | 5 |
| foundationone | 0.27 | 22 |
| find | 0.26 | 54 |
| back | 0.26 | 9 |
| harbor | 0.26 | 24 |
| someone | 0.26 | 7 |
| undergo | 0.26 | 29 |
| patient | 0.26 | 102 |
| present | 0.26 | 2 |
| decline | 0.26 | 18 |
| well | 0.25 | 19 |
| fact | 0.25 | 13 |
| give | 0.25 | 10 |
| stage | 0.25 | 6 |
| movick | 0.25 | 19 |
| somatic | 0.25 | 63 |
| underlie | 0.25 | 20 |
| possibility | 0.25 | 23 |
| show | 0.24 | 41 |
| wish | 0.24 | 11 |
| clinic | 0.24 | 10 |
| carrier | 0.24 | 74 |
| genomic | 0.24 | 33 |
| ovarian | 0.24 | 53 |
| believe | 0.24 | 25 |
| slightly | 0.24 | 4 |
| mutation | 0.24 | 31 |
| absolute | 0.24 | 6 |
| therefore | 0.24 | 28 |
| predisposition | 0.24 | 9 |
| dx | 0.23 | 22 |
| also | 0.23 | 21 |
| base | 0.23 | 20 |
| chemgp | 0.23 | 5 |
| expand | 0.23 | 9 |
| general | 0.23 | 23 |
| consent | 0.23 | 9 |
| estimate | 0.23 | 18 |
| clinical | 0.23 | 8 |
| insurance | 0.23 | 29 |
| recurrent | 0.23 | 30 |
| susceptibility | 0.23 | 16 |
| one | 0.22 | 23 |
| pose | 0.22 | 1 |
| still | 0.22 | 11 |
| breast | 0.22 | 77 |
| chance | 0.22 | 26 |
| finding | 0.22 | 12 |
| inherit | 0.22 | 37 |
| candidate | 0.22 | 5 |
| counseling | 0.22 | 11 |
| nbn | 0.21 | 46 |
| year | 0.21 | 26 |
| regard | 0.21 | 9 |
| highly | 0.21 | 9 |
| result | 0.21 | 31 |
| bexley | 0.21 | 11 |
| better | 0.21 | 5 |
| identfy | 0.21 | 14 |
| include | 0.21 | 48 |
| genetics | 0.21 | 14 |
| stephanie | 0.21 | 12 |
| criterion | 0.21 | 6 |
| pancreatic | 0.21 | 16 |
| population | 0.21 | 15 |
| pathogenic | 0.21 | 46 |
| heterozygous | 0.21 | 23 |
| site | 0.2 | 15 |
| xrcc | 0.2 | 33 |
| tube | 0.2 | 6 |
| prove | 0.2 | 7 |
| amend | 0.2 | 6 |
| family | 0.2 | 44 |
| husband | 0.2 | 7 |
| mcewing | 0.2 | 16 |
| explain | 0.2 | 15 |
| five | 0.19 | 16 |
| less | 0.19 | 12 |
| could | 0.19 | 14 |
| normal | 0.19 | 18 |
| option | 0.19 | 12 |
| history | 0.19 | 31 |
| october | 0.19 | 28 |
| relative | 0.19 | 9 |
| available | 0.19 | 13 |
| frequently | 0.19 | 8 |
| approximately | 0.19 | 37 |
| old | 0.18 | 20 |
| many | 0.18 | 4 |
| nccn | 0.18 | 14 |
| high | 0.18 | 7 |
| model | 0.18 | 19 |
| tumor | 0.18 | 41 |
| variant | 0.18 | 21 |
| personal | 0.18 | 11 |
| majority | 0.18 | 6 |
| sensitivity | 0.18 | 9 |
| bracanalysis | 0.18 | 17 |
| continueduntil | 0.18 | 7 |
| due | 0.17 | 14 |
| parp | 0.17 | 28 |
| need | 0.17 | 3 |
| name | 0.17 | 17 |
| cover | 0.17 | 13 |
| winch | 0.17 | 6 |
| either | 0.17 | 14 |
| toghia | 0.17 | 5 |
| female | 0.17 | 38 |
| genetic | 0.17 | 71 |
| perform | 0.17 | 21 |
| respond | 0.17 | 10 |
| multigene | 0.17 | 7 |
| recommend | 0.17 | 8 |
| note | 0.16 | 18 |
| last | 0.16 | 12 |
| basis | 0.16 | 8 |
| would | 0.16 | 11 |
| genedx | 0.16 | 27 |
| mother | 0.16 | 11 |
| manner | 0.16 | 18 |
| report | 0.16 | 15 |
| provide | 0.16 | 10 |
| surgical | 0.16 | 12 |
| moderate | 0.16 | 3 |
| national | 0.16 | 14 |
| mcewingis | 0.16 | 4 |
| concerned | 0.16 | 11 |
| otherwise | 0.16 | 7 |
| tell | 0.15 | 18 |
| large | 0.15 | 22 |
| develop | 0.15 | 29 |
| condition | 0.15 | 1 |
| iv | 0.14 | 14 |
| gf | 0.14 | 20 |
| talk | 0.14 | 9 |
| assess | 0.14 | 6 |
| current | 0.14 | 21 |
| coverage | 0.14 | 16 |
| associate | 0.14 | 35 |
| previously | 0.14 | 19 |
| hereditary | 0.14 | 26 |
| metastatic | 0.14 | 15 |
| individual | 0.14 | 23 |
| open | 0.13 | 5 |
| cause | 0.13 | 17 |
| carry | 0.13 | 20 |
| today | 0.13 | 15 |
| start | 0.13 | 8 |
| treat | 0.13 | 19 |
| pocket | 0.13 | 4 |
| account | 0.13 | 8 |
| usually | 0.13 | 3 |
| increase | 0.13 | 20 |
| placement | 0.13 | 5 |
| laboratories | 0.13 | 8 |
| use | 0.12 | 9 |
| hboc | 0.12 | 15 |
| letter | 0.12 | 7 |
| medica | 0.12 | 6 |
| familial | 0.12 | 7 |
| cbc | 0.11 | 5 |
| brca | 0.11 | 12 |
| mean | 0.11 | 8 |
| copy | 0.11 | 17 |
| elect | 0.11 | 15 |
| place | 0.11 | 12 |
| strong | 0.11 | 7 |
| second | 0.11 | 3 |
| factor | 0.11 | 2 |
| biopsy | 0.11 | 26 |
| discuss | 0.11 | 31 |
| disease | 0.11 | 10 |
| monitor | 0.11 | 3 |
| dominant | 0.11 | 6 |
| criteria | 0.11 | 17 |
| olaparib | 0.11 | 8 |
| breastnext | 0.11 | 1 |
| significant | 0.11 | 3 |
| meet | 0.1 | 19 |
| woman | 0.1 | 26 |
| child | 0.1 | 8 |
| policy | 0.1 | 1 |
| another | 0.1 | 4 |
| seizert | 0.1 | 2 |
| probability | 0.1 | 6 |
| information | 0.1 | 7 |
| additionally | 0.1 | 3 |
| mayo | 0.09 | 2 |
| type | 0.09 | 17 |
| picc | 0.09 | 4 |
| visit | 0.09 | 15 |
| blood | 0.09 | 10 |
| receive | 0.09 | 9 |
| discover | 0.09 | 6 |
| difficult | 0.09 | 9 |
| rtc | 0.08 | 3 |
| man | 0.08 | 4 |
| term | 0.08 | 3 |
| gerl | 0.08 | 3 |
| screen | 0.08 | 2 |
| gemzar | 0.08 | 5 |
| however | 0.08 | 7 |
| medicare | 0.08 | 12 |
| lynparza | 0.08 | 12 |
| inhibitor | 0.08 | 29 |
| typically | 0.08 | 4 |
| autosomal | 0.08 | 6 |
| prevalence | 0.08 | 1 |
| ii | 0.07 | 5 |
| pay | 0.07 | 15 |
| male | 0.07 | 5 |
| time | 0.07 | 10 |
| april | 0.07 | 8 |
| refer | 0.07 | 3 |
| specimen | 0.07 | 3 |
| paternal | 0.07 | 7 |
| endometrial | 0.07 | 6 |
| ca | 0.06 | 3 |
| via | 0.06 | 3 |
| fam | 0.06 | 8 |
| face | 0.06 | 8 |
| july | 0.06 | 7 |
| keri | 0.06 | 2 |
| take | 0.06 | 6 |
| first | 0.06 | 5 |
| might | 0.06 | 5 |
| remain | 0.06 | 5 |
| around | 0.06 | 3 |
| whether | 0.06 | 2 |
| hodgkin | 0.06 | 1 |
| consider | 0.06 | 4 |
| lifetime | 0.06 | 12 |
| syndrome | 0.06 | 5 |
| treatment | 0.06 | 12 |
| rucaparib | 0.06 | 21 |
| adenocarcinoma | 0.06 | 3 |
| yet | 0.05 | 5 |
| gyn | 0.05 | 6 |
| age | 0.05 | 8 |
| self | 0.05 | 1 |
| word | 0.05 | 2 |
| role | 0.05 | 7 |
| member | 0.05 | 9 |
| affect | 0.05 | 4 |
| cousin | 0.05 | 6 |
| several | 0.05 | 2 |
| feature | 0.05 | 6 |
| multiple | 0.05 | 7 |
| carefully | 0.05 | 1 |
| interested | 0.05 | 8 |
| approximate | 0.05 | 4 |
| informative | 0.05 | 5 |
| two | 0.04 | 7 |
| case | 0.04 | 12 |
| leave | 0.04 | 3 |
| axilla | 0.04 | 4 |
| likely | 0.04 | 6 |
| assist | 0.04 | 6 |
| sporadic | 0.04 | 1 |
| encompass | 0.04 | 4 |
| screening | 0.04 | 2 |
| responsible | 0.04 | 2 |
| men | 0.03 | 5 |
| health | 0.03 | 2 |
| depend | 0.03 | 4 |
| network | 0.03 | 4 |
| elevate | 0.03 | 2 |
| briefly | 0.03 | 1 |
| primary | 0.03 | 6 |
| etiology | 0.03 | 1 |
| diagnose | 0.03 | 9 |
| relation | 0.03 | 3 |
| mckinney | 0.03 | 1 |
| followup | 0.03 | 6 |
| education | 0.03 | 5 |
| guideline | 0.03 | 6 |
| full | 0.02 | 3 |
| relate | 0.02 | 4 |
| reflex | 0.02 | 2 |
| degree | 0.02 | 2 |
| counsel | 0.02 | 2 |
| detection | 0.02 | 3 |
| nephrology | 0.02 | 2 |
| peritoneal | 0.02 | 1 |
| unfortunately | 0.02 | 1 |
| state | 0.01 | 2 |
| point | 0.01 | 1 |
| specify | 0.01 | 2 |
| lymphoma | 0.01 | 2 |
| heritable | 0.01 | 2 |
| recommendation | 0.01 | 2 |
| list | 0 | 0 |
| make | 0 | 0 |
| week | 0 | 0 |
| topic | 0 | 0 |
| north | 0 | 0 |
| three | 0 | 0 |
| compare | 0 | 1 |
| specific | 0 | 0 |
| threefold | 0 | 1 |
| malignancy | 0 | 1 |
| management | 0 | 0 |
| epithelial | 0 | 0 |
| prevention | 0 | 0 |
